# P-854. Determining whether presence of Lower Urinary Tract Infection Symptoms is Associated with Better Graft Outcomes in Kidney Transplant Recipients with Bacteremia from a Urinary Source

**DOI:** 10.1093/ofid/ofae631.1046

**Published:** 2025-01-29

**Authors:** Maja Wichhart Donzo, Rebecca Anderson, Emily Eichenberger

**Affiliations:** Emory University School of Medicine, Atlanta, Georgia; Emory University School of Medicine, Atlanta, Georgia; Emory School of Medicine, Atlanta, Georgia

## Abstract

**Background:**

Bloodstream infection is a feared complication of bacteriuria in kidney transplant recipients (KTR). We recently found that among KTR with blood stream infection from a urinary source (BSIU), only 28.9% had experienced lower urinary tract infection (UTI) symptoms prior to or upon presentation for their bacteremia. We hypothesized that presence of lower UTI symptoms may lead to earlier recognition of BSIU and therefore better outcomes.

**Figure:**
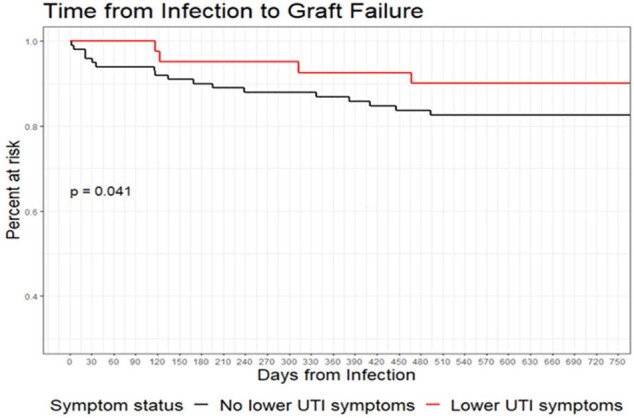

**Methods:**

Patients who underwent a kidney transplant at our institution between 01/2010-09/2022 with BSIU were eligible for inclusion. BSIU was defined as KTR with concordant organisms growing in blood and urine cultures. For subjects with >1 episode of BSIU, only the first episode was included for analysis. KTR with BSIU were classified into one of two groups: presence of lower UTI symptoms or absence of lower UTI symptoms. Lower UTI symptoms were defined as presence of urinary frequency, urgency, dysuria, or suprapubic pain.

Continuous and categorical variables were compared using Wilcoxon-rank sum and fisher exact tests as appropriate. Time to graft failure and acute cellular rejection were analyzed with Kaplan Meier curves and compared using log rank tests.

**Results:**

Among 142 patients who presented with BSIU, 41 (28.9%) presented with lower UTI symptoms. Relative to BSIU without UTI symptoms, BSIU with lower UTI symptoms were less likely to have a history of diabetes induced renal failure (20% vs 43%, P=0.012), and less likely to have a had acute cellular rejection preceding infection (7.3% vs 27%, P=0.012). Race, gender, recipient age at transplant, donor type, and immunosuppression regimen did not differ between the two groups. 90-day mortality among BSIU was 0% for those with lower UTI symptoms versus 4% for those without symptoms (P=0.324). Time from BSIU to acute cellular rejection did not differ by symptom status (P = 0.99). Overall time to graft failure was longer in BSIU with UTI symptoms (P=0.041).

**Conclusion:**

Longer time to graft failure was noted among BSIU who presented with lower UTI symptoms. Larger studies are needed to validate these findings and investigate how we can use clinical data and symptomatology to predict outcomes for KTR with BSIU.

**Disclosures:**

**All Authors**: No reported disclosures

